# Inflammasome Activation Induced by a Snake Venom Lys49-Phospholipase A_2_ Homologue

**DOI:** 10.3390/toxins12010022

**Published:** 2019-12-31

**Authors:** Charles Nunes Boeno, Mauro Valentino Paloschi, Jéssica Amaral Lopes, Weverson Luciano Pires, Sulamita da Silva Setúbal, Jaína Rodrigues Evangelista, Andreimar Martins Soares, Juliana Pavan Zuliani

**Affiliations:** 1 Laboratório de Imunologia Celular Aplicada à Saúde, Fundação Oswaldo Cruz, FIOCRUZ Rondônia, 76812-245 Porto Velho-RO, Brazil; charlesboeno_chls@hotmail.com (C.N.B.); paloschimauro@gmail.com (M.V.P.); jessicah.amaral@gmail.com (J.A.L.); pirescell@gmail.com (W.L.P.); sulamitasetubal@gmail.com (S.d.S.S.); jaina83.jr@gmail.com (J.R.E.); 2 Centro de Estudos de Biomoléculas Aplicadas à Saúde (CEBio), Fundação Oswaldo Cruz, FIOCRUZ Rondônia e Departamento de Medicina, Universidade Federal de Rondônia, UNIR, 76812-245 Porto Velho-RO, Brazil; andreimarsoares@gmail.com; 3 Centro Universitário São Lucas, UniSL, 76805-846 Porto Velho, RO, Brazil

**Keywords:** snake venom, Lys49-PLA_2_, muscle, inflammasome

## Abstract

Background: Snake venom phospholipases A_2_ (PLA_2_s) have hemolytic, anticoagulant, myotoxic, oedematogenic, bactericidal, and inflammatory actions. BthTX-I, a Lys49-PLA_2_ isolated from *Bothrops jararacussu* venom, is an example of Lys49-PLA_2_ that presents such actions. NLRP3 is a cytosolic receptor from the NLR family responsible for inflammasome activation via caspase-1 activation and IL-1β liberation. The study of NLRs that recognize tissue damage and activate the inflammasome is relevant in envenomation. Methods: Male mice (18–20 g) received an intramuscular injection of BthTX-I or sterile saline. The serum was collected for creatine-kinase (CK), lactate dehydrogenase (LDH), and interleukin-1β (IL-1β) assays, and muscle was removed for inflammasome activation immunoblotting and qRT-PCR expression for nucleotide and oligomerization domain, leucine-rich repeat-containing protein family, pyrin-containing domain 3 receptor (NLRP3) inflammasome components. Results: BthTX-I-induced inflammation and myonecrosis, shown by intravital microscope, and LDH and CK release, respectively. Mouse treatment with A438079, a P2X7 receptor antagonist, did not modify these effects. BthTX-I induced inflammasome activation in muscle, but P2X7R participation in this effect was not observed. Conclusion: Together, the results showed for the first time that BthTX-I in gastrocnemius muscle induces inflammation and consequently, inflammasome activation via NLRP3 with caspase-1 activation and IL-1β liberation.

## 1. Introduction 

Inflammasomes are multiprotein complexes present in the cytosol of immune cells. These complexes sense and respond to pathogen infection or tissue injury, and one of the most extensively studied inflammasomes is the NLRP3 (nucleotide and oligomerization domain, leucine-rich repeat-containing protein family, pyrin-containing domain 3 receptor) [1]. Following the assembly of NLRP3 inflammasome, intracellular caspase-1 (cysteine-dependent aspartate-directed protease-1) is activated to catalyze pro-interleukin-1β (pro-IL-1β) cleavage into mature IL-1β [1]. As a canonical activator of NLRP3 inflammasome, ATP can be released from both host tissue injury [2,3] and bacterial cells during infection [4].

Notably, IL-1β cytokine release, a pro inflammatory mediator liberated as a consequence of leukocyte activation that promotes a variety of immunologic effects, depends on pyroptosis [5]. Pyroptosis precedes caspase-1 activation and is characterized by a rapid cell swelling and membrane rupture, leading to the release of intracellular contents. During the pyroptosis process, several channels or pores can be opened or formed in the cell membrane, enabling the release of intracellular contents [6]. Mechanistically, ATP induces inflammasome activation mainly through its action on cell membrane P2X7 receptors [7,8]. After ATP engagement, P2X7 receptors form a nonselective cation channel for K^+^ ion efflux. If P2X7 receptors are persistently activated, they may further recruit pannexin-1 to form pores that promote IL-1β release [9,10,11] and induce cell death.

Therefore, inflammasome activation is an inflammatory response that occur during infection or injury that can carry the host to eliminate the pathogens or repair the damaged tissues by recruiting various inflammatory immune cells. However, sustained inflammasome activation by endogenous danger signals released from damaged cells may aggravate the inflammatory process in sterile inflammatory disorders [12,13,14].

Venoms from Viperidae snakes contain Group II secreted phospholipases A_2_ (sPLA_2_s), which share structural features with sPLA_2_s present in inflammatory exudates in mammals [15,16]. In *Bothrops jararacussu* snake venom, a catalytically inactive variant, which has a lysine (Lys) residue instead of aspartate (Asp) residue at position 49, was characterized [17,18,19]. This modification, along with other changes in the calcium-binding loop, prevents an effective calcium binding and consequently, is responsible for the absence of the enzymatic activity described in these PLA_2_ variants or homologue [20,21,22,23]. In addition to myotoxicity, these Lys-PLA_2_s induce inflammatory events and release important inflammatory mediators under both in vivo and in vitro experimental conditions [23,24,25,26,27].

Direct cytotoxicity, leading to necrosis, represents one output of envenomation. The entry of snake venom components, particularly sPLA_2_s, into tissues affects resident cells in different ways. However, other cells may be reached by noncytotoxic concentrations of snake venom components; in these cases, other cellular responses, distinct from cell death, may develop, and they may contribute to the overall tissue alterations observed. The interference of snake venom PLA_2_ in inflammasome activation in muscles is still unknown and can contribute to the severe local effects observed in snakebites. The present study was, therefore, developed to evaluate the effects of snake venom Lys49-PLA_2_ homologues on inflammasome activation in vivo in gastrocnemius muscle.

## 2. Results

### 2.1. BthTX-I Induced Inflammation and Myonecrosis in Mouse Gastrocnemius Muscle

**Figure 1:**
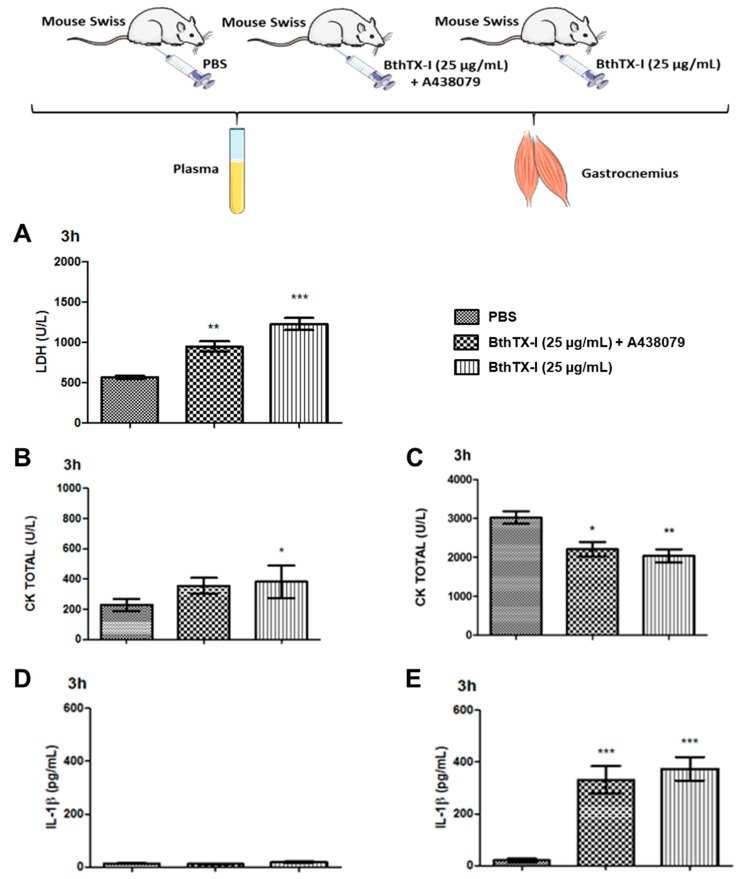
Release of LDH and CK by gastrocnemius muscle induced by BthTX-I. Groups of mice were injected with PBS (control) or BthTX-I (25 µg/mL) treated with or without A438079 (an antagonist of P2X7 receptor), 80 µmol/kg, intraperitoneal route, 30 min before BthTX-I injection. LDH (**A**), serum CK (**B**), residual muscle CK (**C**), serum IL-1β (**D**), and muscle IL-1β (**E**) were analyzed after 3 h of toxin or PBS inoculation, as detailed in Materials and Methods. Values represent the mean ± SEM of five animals (n = 5). * *p* < 0.05, ** *p* < 0.001, *** *p* < 0.0001 when compared with control (ANOVA).

To assess the inflammatory and myotoxic reaction induced by BthTX-I in vivo, LDH and CK levels, respectively, were determined in mice serum. Data obtained showed that BthTX-I induced a significant release of both mediators, LDH in plasma (Figure 1A) and CK (Figure 1B), 3 h after its injection in mouse gastrocnemius muscle compared with control. These liberated mediators confirm the myotoxic action of the BthTX-I. Moreover, the residual muscular tissue CK was determined in the gastrocnemius muscles (Figure 1C), and this showed that the majority of CK was liberated to the circulation, confirming the BthTX-I myotoxic action. With respect to P2X7 receptor participation in these effects it was observed that this treatment reduced the CK liberation from mice muscles.

### 2.2. IL-1β Release by Injury in Muscle Gastrocnemius Induced by BthTX-I

Literature shows that following the assembly of inflammasome, intracellular caspase-1 is activated to catalyze pro-interleukin-1β (pro-IL-1β) cleavage into mature IL-1β [1]. Considering this, we investigated the IL-1β liberation in muscle tissue inoculated with BthTX-I. The results obtained showed that BthTX-I did not induced IL-1β release to serum after 3 h of its inoculation in gastrocnemius muscles (Figure 1D). Mice treated with A438079, a P2X7 receptor antagonist, and inoculated with BthTX-I did not modify this parameter compared with the mice group without treatment but inoculated with BthTX-I. On the other hand, it was observed that IL-1β was stocked in mice muscles stimulated with BthTX-I, as shown in Figure 1E. 

### 2.3. IL-1β Release by Injury in Muscle Gastrocnemius Induced by BthTX-I

**Figure 2:**
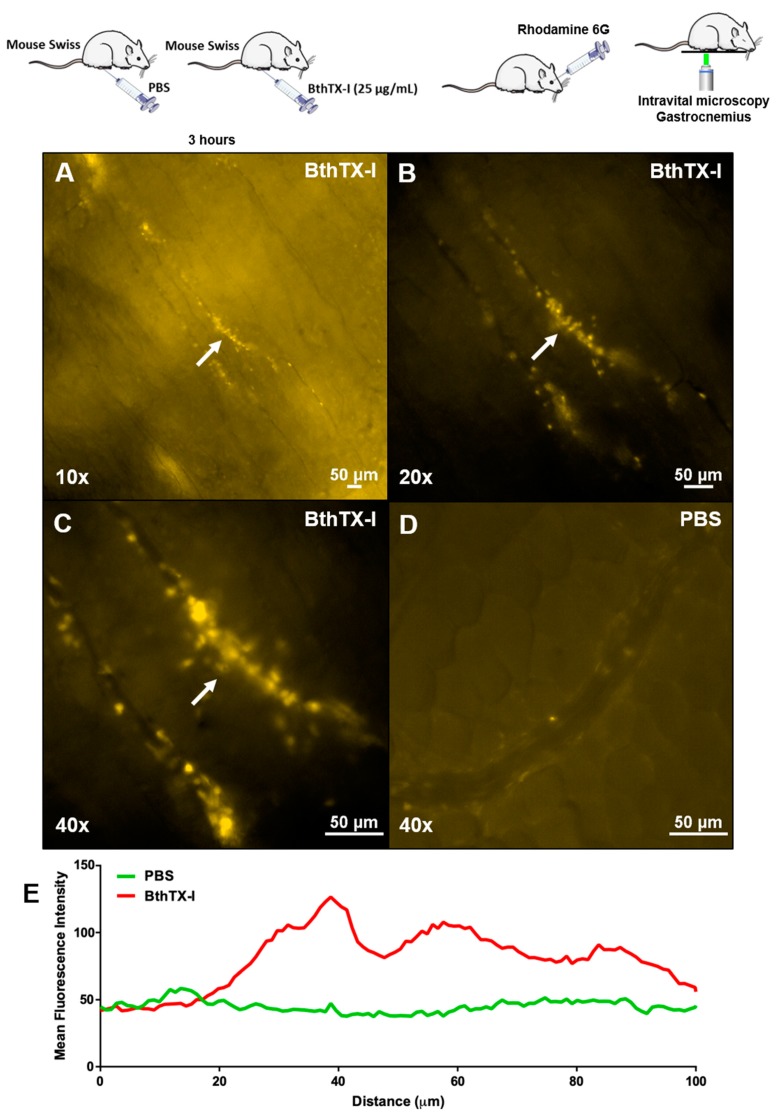
Intravital images of gastrocnemius muscle induced by BthTX-I. Groups of mice were injected with PBS (control) or BthTX-I (25 µg/animal). Intravital microscopy was analyzed after 3 h of toxin or PBS inoculation as detailed in Materials and Methods. Leukocyte influx induced by BthTX-I with 10× magnificence (**A**), BthTX-I with 20× magnificence (**B**), BthTX-I with 40 X magnificence (**C**), and sterile PBS (control) (**D**). The graph (**E**) represents the mean fluorescence intensity of the images. Representation of one animal control and one animal inoculated with BthTX-I (n = 2).

The inflammatory infiltrate in gastrocnemius muscle was visualized by intravital microscopy. Mice inoculated with sterile PBS (controls mice) in gastrocnemius muscle showed macroscopically absence of edema and hemorrhage. Intravital microscope images of control mice showed absence of leukocyte influx into the surrounding tissue and normal blood circulation without blood stasis parameters or interaction of cells with the endothelium vessels, as shown in (Figure 2D) and Supplementary Video (S1). The same procedure was conducted with animals inoculated with BthTX-I in the gastrocnemius muscle. Macroscopically, this protein induced an extensive muscle edema and an important bleeding at the site of inoculation committing the entire mouse lower member (leg and paw). Intravital microscope images showed leukocyte influx into muscle, blood stasis, leukocytes interacting with the endothelium and performing migration to the tissue induced by BthTX-I (Figure 2A–C and Supplementary Video (S2)).

### 2.4. BthTX-I Induced Inflammasome Activation in Mouse Gastrocnemius Muscle

**Figure 3:**
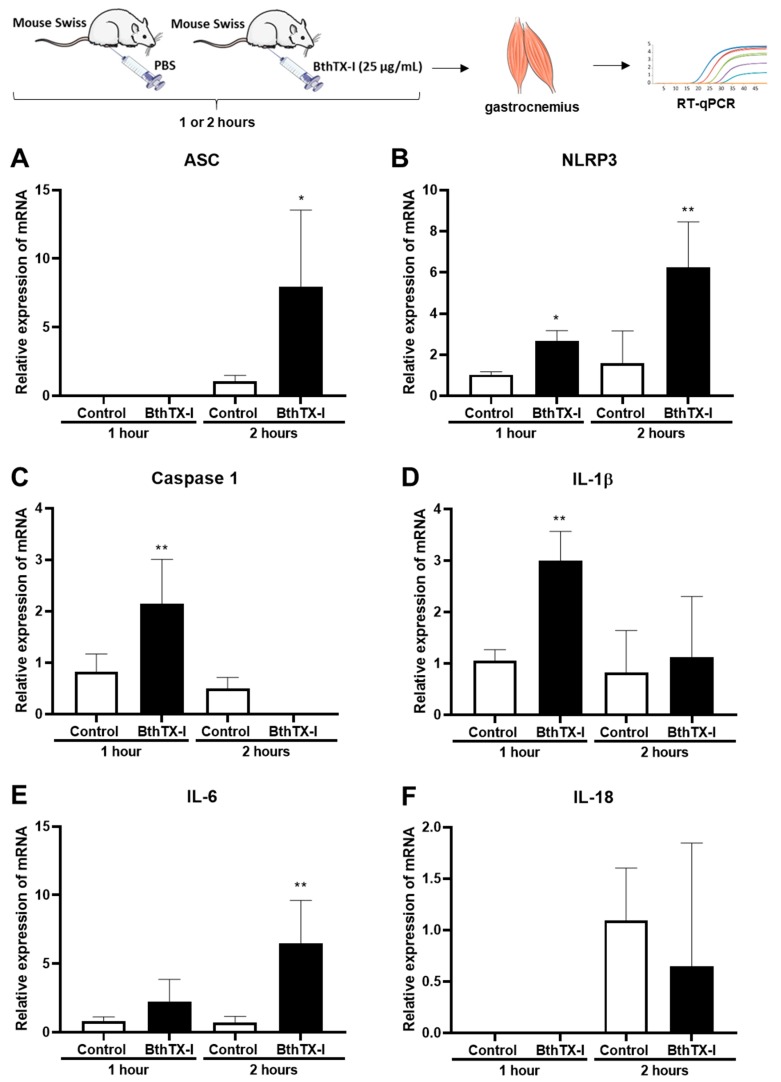
Gene expression of NLRP3 inflammasome complex. Swiss mice were inoculated with PBS or BthTX-I into the gastrocnemius muscle. After 1 or 2 h, the muscles were removed and processed for RNA extraction and for inflammasome NLRP3 components gene expression. Gene expression of ASC protein (associated CARD domain) (**A**), NLRP3 (NOD-, LRR-, and pyrin domain-containing protein 3 receptor) (**B**), Caspase 1 Effector Protein (**C**), pro-inflammatory cytokines IL-1β (**D**), IL-6 (**E**), and IL-18 (**F**). Values represent the mean ± SEM of three animals (n = 3). * *p* < 0.05, ** *p* < 0.001 when compared with control (ANOVA).

Inflammasome activation represents an inflammatory response during tissue injury that can benefit the host to clear the pathogens or to repair the injured tissues by recruiting inflammatory immune cells. Thus, from mice muscles inoculated with BthTX-I or sterile PBS, mRNA were extracted with Trizol Reagent and used for qRT-PCR, using specific gene primers for the components of inflammasome NLRP3: ASC, NLRP3, Caspase-1, IL-1β, IL-6, IL-18, P2X7R, and β-actin, the normalizing gene. According to Figure 3B, 3C and 3D NLRP3, Caspase-1, and IL-1β gene expression was observed in animals injected after 1 h. After this time, it was not observed the Caspase-1 gene expression in muscles inoculated with BthTX-I significantly different from control animals. In addition, after 2 h of BthTX-I injection in muscles, it was observed an increment of ASC, NLRP3, and IL-6 gene expressions significantly different from that of control animals (Figure 3A,B,E). However, there was no observed difference between both groups for IL-18 gene expression (Figure 3F). With respect to the P2X7R, the gene expression for P2X7R was not expressed in these studied time intervals (data not shown). 

**Figure 4:**
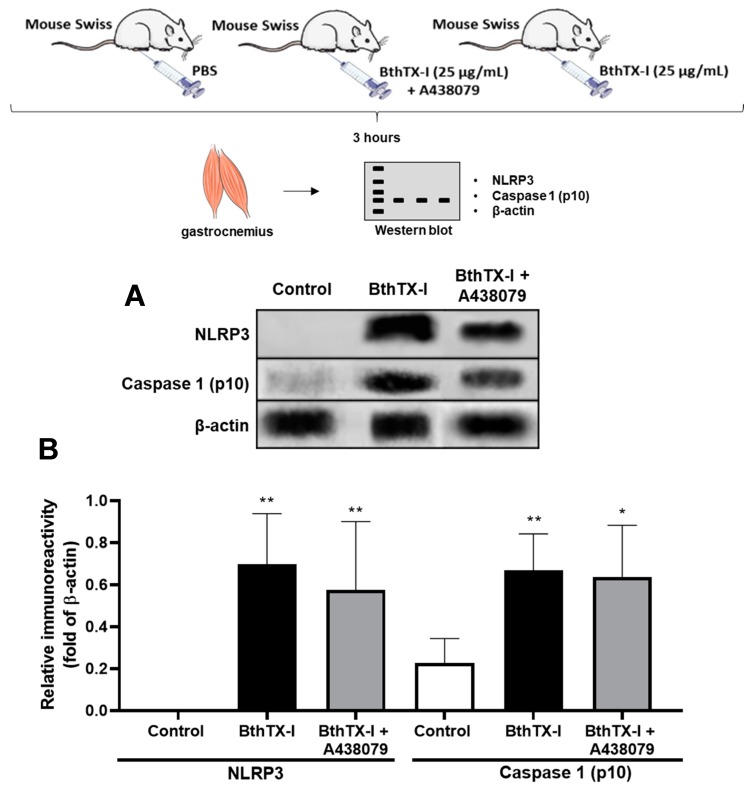
Protein expression of Swiss mice divided into three groups: inoculated with PBS, BthTX-I, and one group inoculated with BthTx-I and treated with P2X7R receptor inhibitor via intraperitoneal (i.p.) injection (30 min after BthTX-I injection into the gastrocnemius muscle). After 3 h of these injections, the muscles were removed and processed for NLRP3 inflammasome components protein expression. *Western blot* of NLRP3 inflammasome components expressed in gastrocnemius muscle: NLRP3 receptor, Caspase 1 (p10) from BthTX-I-inoculated, A438079-treated and BthTX-I-inoculated groups, and control PBS (**A**). *Western blot* densitometry analysis (**B**). Values represent the mean ± SEM of three animals (n = 3). * *p* < 0.05, ** *p* < 0.001 when compared with control (ANOVA).

In addition, it was observed that Caspase-1 and NLRP3 protein expression in mice inoculated after 3 h with BthTX-I on muscle gastrocnemius (Figure 4A,B) was different from that of control animals that received sterile PBS in muscles. The mice treatment with P2X7R antagonist (A438079) did not modify this parameter. Moreover, as can be seen in Figure 1E, mice treated with A438079 and injected with BthTX-I showed IL-1β release from muscle gastrocnemius, but this cytokine was not observed in plasma mice (Figure 1D). These findings evidenced the ability of BthTX-I to activate inflammasome in mouse muscle.

## 3. Discussion

*Bothrops jararacussu* snake venom contains different toxins; PLA_2_s are the majority. These enzymes act on muscle, inducing damage to this tissue; the myotoxicity. The hallmark of experimental envenomation induced by snake venom PLA_2_ is the CK release to the plasma, as described by Gutierrez and Ownby [28], Gutierrez et al. [29], and Lomonte and Gutierrez [30]. Present results showed that BthTX-I, a Lys49 snake venom PLA_2_, after 3 h of inoculation in gastrocnemius muscle, cytotoxic and myotoxic reactions were induced in experimentation animals, observed by significant LDH levels release, especially after the loss of the integrity of the cell membrane, and CK release after muscle damage. Melo et al. [31] and Veronese et al. [32] showed CK release in experimental animals after BthTX-I administration, confirming the data currently obtained, corroborating with the present results. 

Moreover, besides myotoxicity, PLA_2_ also induces an inflammatory process characterized by an intense leukocyte influx. The inflammatory effects, most of them, are associated with the production of mediators involved in many biological processes, such as cytokines and lipid mediators; prostaglandins, thromboxane, and leukotrienes [33,34]. In the present study, using intravital microscope images it was possible to observe a leukocyte influx into muscle tissue (Supplementary Video S1 and S2), confirming the presence of immune cells in the muscle gastrocnemius inoculated with BthTX-I.

Additionally, literature shows that myofibers undergoing necrosis release danger-associated molecular patterns (DAMPs) that activate innate immune receptors, resulting in cytokines and chemokines release and a subsequent recruitment of inflammatory cells that can remove necrotic debris and facilitate muscle regeneration [35]. To this end, besides myotoxicity and cytotoxicity, the possibility of BthTX-I, a Lys49-PLA_2_ homologue, to induce IL-1β release from gastrocnemius muscle was investigated. The obtained results showed that BthTX-I induced IL-1β release by muscle tissue after 3 h of administration, which was different from control animals. Supporting this, Jossten et al. [36] described that IL-1β precursor is not present in health but inducible because macrophages do not constitutively express mRNA for IL-1β. 

Regarding the participation of P2X7 receptor in inflammatory response, it should be mentioned that the literature shows that ATP released by cells that have suffered damage contributes to increase inflammatory response through the synthesis and release of PGE_2_ [37] via P2X7 receptors [38]. However, in the present study, BthTX-I-induced cytotoxicity and myotoxicity in animals treated with or without the A438079, a P2X7 receptor antagonist, showed that this receptor is not involved in the observed effects. There is no data in the literature to date showing the involvement of P2X7 receptors on cytotoxicity and myotoxicity actions by toxins isolated from snake venoms, particularly PLA_2_. Other purinergic receptors may be involved in this effect and will be studied to clarify this mechanism.

In addition, there was no significant difference between animals treated or not with the P2X7 receptor antagonist on IL-1β release. Rawat et al. [39] showed that normal primary skeletal muscle cells are able to induce IL-1β release when treated with lipopolysaccharide, ATP, and P2X7 receptor agonist stimuli. This study suggests that immune cells present in muscle and even muscle cells may actively participate in the activation of the NLRP3 inflammasome. However, IL-1β release to the circulation was not observed after BthTX-I experimental envenomation treated and nontreated with P2X7 receptor agonist compared with control animals. 

Several studies report that Lys49-PLA_2_ homologue promotes the ATP release from myotubes by an unknown mechanism [40,41,42]. Recently, Zhang et al. [43] showed that a PLA_2_ isolated from *B. moojeni* venom activates a subpopulation of somatosensorial neurons that contribute to pain, to induce the ATP release via pannexin hemichannels, which activates P2X3 and P2X2 purinergic receptors, causing acute pain, tissue inflammation, and hyperalgesia; clarifying the toxin mechanism of action in the painful phenomenon.

It is worth mentioning the presence of leukocytes after intramuscular injection of sPLA_2_s in the literature [34]. It was also showed that BthTX-I has pro-inflammatory action, characterized by swelling, recruitment of leukocytes, and cell activation with an increase of phagocytosis and release of lipid droplets and mediators, such as leukotrienes [44,45,46,47,48]. Some inflammatory cytokines and a series of lipid and inflammatory mediators liberated by activated leukocytes can cause tissue damage [49].

Considering that ATP is a DAMP molecule and can activate the inflammasome/NLRP3 [49,50,51], and NLRP3, via adaptor protein ASC recruitment, recruits and activates the pro-caspase-1 and this suffers proteolytic cleavage, becoming active and mediating the processing and activation of pro-inflammatory cytokines such as IL-1β and IL-18, studies were conducted to verify if BthTX-I was able to activate the inflammasome NLRP3 complex. qRT-PCR results showed that BthTX-I induced NLRP3, ASC, Caspase-1, and IL-1β gene expression, showing that this PLA_2_ is able to activate NLRP3 inflammasome. The specific genes expression that coordinates the formation of these proteins precedes their translation, therefore, the presence of mRNA is necessary at shorter times than those for protein expression. Thus, the inflammasome NLRP3 gene expression in the gastrocnemius muscle was determined after 1 and 2 h of BthTX-I inoculation.

Moreover, the obtained protein expression results showed NLRP3 and Caspase-1 were significantly induced in BthTX-I experimental group compared with control group, confirming the inflammasome NLRP3 complex activation.

**Figure 5:**
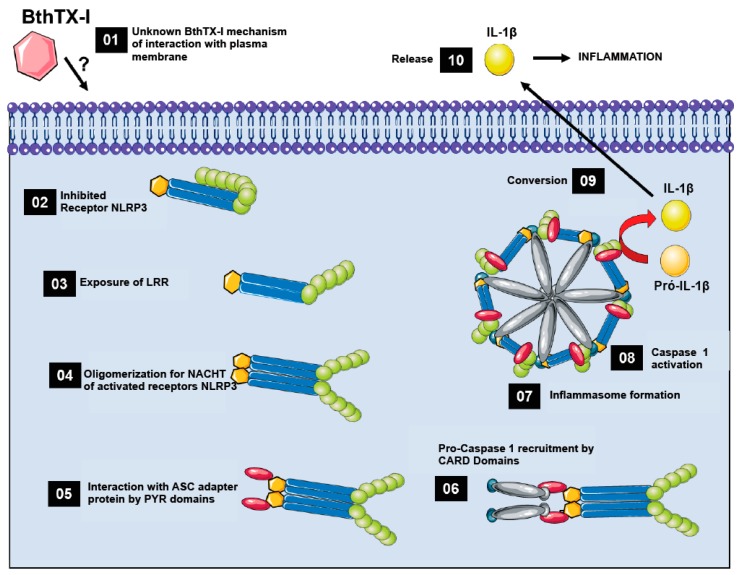
Proposed BthTX-I mechanism of action on muscle. (**01**) BthTX-I interaction by an unknown manner, (**02**) NLRP3 receptor (NOD-, LRR- and pyrin domain-containing protein 3) with inhibited leucine repetitions, (**03**) tail exposure of leucine repeats for binding to activator molecules, DAMPs or PAMPs, (**04**) NACHT domain receptor oligomerization, (**05**) ASC adapter protein recruitment by interactions with pyrin domains, (**06**) Pro-caspase 1 protein recruitment by CARD domains and receptor protein complex, adapter protein, and effector protein formation, (**07**) formation of the high-molecular-weight inflammasome NLRP3 complex with oligomerization of activated components, (**08**) activation of Protein Caspase 1 initiating cleavage of inflammatory cytokine IL-1β, (**09**) conversion of immature pro-IL-1β to IL-1β activated by action of the NLRP3 Inflammasome complex, (**10**) IL-1β release.

With the obtained data it was possible to propose a BthTX-I mechanism of action (Figure 5). BthTX-I interacts with the cellular membrane by an unknown receptor. NLRP3 are found in an inhibited form in the cellular cytoplasm, and after the release of signaling molecules such as DAMPs from cellular damage, the NLRP3 are activated and the protein interaction is initiated, leading to the formation of the inflammasome complex. Activated NLRP3 interacts with ASC adapter protein and pro-caspase-1 protein. Activation of this complex leads to caspase-1 activation and pro-inflammatory IL-1β protein release that contributes to the inflammatory process.

## 4. Conclusions

Together, the results allow to conclude that BthTX-I evoked in in vivo studies the inflammatory effect and the cellular damage through the release of LDH and CK, respectively, besides the inflammasome activation with caspase-1 activation and IL-1β production in muscle tissue. These results are unpublished and contribute to the knowledge of BthTX-I mechanisms of action on inflammatory and myotoxic reactions. 

## 5. Materials and Methods

### 5.1. Chemicals and Reagents

3,3′,5,5′-Tetramethylbenzidine (TMB), bovine serum albumine (BSA), ATP, protease and phosphatase inhibitors, hydrogen peroxide, bicinchoninic acid (BCA) protein assay kit, anti-β-actina (A1978-200UL) isotype mouse, and 3,3′diaminobenzidine (DAB) were purchased from Sigma Aldrich (Sant Louis, MO, USA). Goat anti-mouse antibody conjugated to horseradish peroxidase (A90-116P) was purchased from Bethyl Laboratories (Montgomery, TX, USA). Mouse IL-1β/IL-1F2 kit *DuoSet* ELISA was purchased from R&D Systems (Oxon, UK). Anti-caspase-1 p10 (AG-20B-0044-C100) and anti-NLRP3 (AG-20B-0014), both from mouse isotype, were purchased from Adipogen Life Sciences (San Diego, CA, USA). PVDF Membrane was purchased from Millipore (Darstadt, Germany). P2X7 receptor antagonist and A438079 were purchased from Tocris Bioscience (Bristol, UK). CK-NAC Liquiform and LDH Liquiform were purchased from Labtest (Minas Gerais, Brazil). Reagents were obtained from Merck (Darmstadt, Germany). Trizol^®^ Reagent and SuperScript III Reverse Transcriptase were purchased from Thermo Fisher Scientific (Waltham, MA, USA). iTaq Universal SYBR® Green Supermix was purchased from Bio-Rad (Hercules, CA, USA).

### 5.2. Animals

Male Swiss mice (18–20 g) were used. These animals were housed in temperature-controlled rooms and received water and food ad libitum until used. These studies were approved by the Ethics Committee on the use of animals from Oswaldo Cruz Foundation—Rondônia (protocol nº 2018/04), approved on 3 May 2018, in accordance with the procedures laid down by the Universities Federation for Animal Welfare. 

### 5.3. Phospholipases A_2_

Lys49-PLA_2_ homologue (BthTX-I) was purified from *Bothrops jararacussu* snake venom according to Andrião-Escarso et al. [52] at Centro de Estudos de Biomoléculas Aplicadas à Saúde (CEBio), Fundação Oswaldo Cruz, FIOCRUZ Rondônia. Homogeneity was demonstrated by SDS–polyacrylamide gel electrophoresis, run under reducing conditions [53].

### 5.4. Inflammatory and Myotoxic Reactions Induced in Gastrocnemius Muscle by BthTX-I

Two groups of five mice (18–20 g) each received an intramuscular injection of 50 µL, in the right thigh, of sterile phosphate-buffered saline (PBS, 14 mM NaCl, 2 mM NaH_2_PO_4_H_2_O, 7 mM Na_2_HPO_4_12H_2_O) pH 7.2 or 50 µL of BthTX-I (25 µg/mL) dissolved in sterile PBS, according to Souza et al. [54]. The third group received 50 µL of BthTX-I (25 µg/mL) dissolved in sterile PBS, but 30 min before of this inoculation the animals were treated with A438079 (an antagonist of P2X7 receptor; 80 µmol/kg, via intraperitoneal injection [55]). After 3 h, mice were euthanized by cervical dislocation. Blood samples throughout this study were obtained from the orbital plexus. After clotting, plasma was separated by centrifugation and the following assays were performed: in plasma: creatine kinase (CK); lactate dehydrogenase (LDH), using CK-NAC Liquiform and LDH Liquiform kits, respectively; and IL-1β levels. The gastrocnemius muscle was removed and homogenized at 20,500–30,000 rpm with 200 µL of PBS, by automatic homogenizer. After centrifugation at 1500 rpm for 5 min, the supernatant was collected to determine CK and IL-1β levels using, respectively, the kits mentioned above. The remaining pellets were suspended in RIPA buffer (Tris-HCl 100 mM, pH 7,6, NaCl 200 mM, CaCl_2_ 100 mM and 1% Triton X-100), containing phosphatase and protease inhibitors (1/100), and homogenized for caspase-1, NLRP3, and β-actin immunoblotting.

### 5.5. Intravital Microscopy of Gastrocnemius Muscle Inoculated with BthTX-I

Two groups of three mice (18–20 g) each received an intramuscular injection of 50 µL, in the right thigh, of sterile PBS pH 7.2 or 50 µL of BthTX-I (25 µg/mL) dissolved in sterile PBS, according to Souza et al. [54]. After 3 h of these injections, the animals were anesthetized with a mixture of ketamine hydrochloride (80 mg/kg) and xilazine hydrochloride (10 mg/kg) via intraperitoneal (i.p.) injection. After anesthesia, the gastrocnemius muscle was exposed for microscope visualization. For staining muscle tissues and circulating leukocytes, rhodamine 6G (1mg/mL) was inoculated into orbital plexus to observe the cell infiltrate. Microscope images were acquired under Nikon Eclipse T*i* microscope using Cy3 filter in different increments. Microscope images were analyzed using the software NIS-Elements.

### 5.6. Immunoblotting

The remaining pellets, resuspended in RIPA buffer with protease and phosphatase inhibitors, from Section 5.4, were used for Western blotting. Immunoblotting was performed using monoclonal antibodies targeting murine anti-β-actin, anti-caspase-1, and anti-NLRP3. For β-actin, caspase-1, and NLRP3 determinations, total protein extracts were prepared, resolved by 10% SDS/PAGE, and transferred onto a PVDF membrane (Hybond, Amersham Pharmacia Biotech, Little Chalfont, UK). Immunoblotting was performed using monoclonal antibodies to the anti-β-actin, anti-caspase-1, and anti-NLRP3. Blots were developed with 3,30-diaminobenzidine tablets and hydrogen peroxide. The relative immunoreactivity bands of three independent experiments were quantified by densitometry using Image Studio Lite Ver 5.2 (LI-COR, Lincoln, NE, USA). The mean densitometry values of anti-caspase-1 and anti-NLRP3 were divided by the mean densitometry values of respective β-actin values to show the relative expression of each protein as a ration mean of the protein/β-actin.

### 5.7. Interleukin-1β (IL-1β) Quantification

For this assay, the samples obtained from the inoculated (right) muscle and plasma from mice, obtained as described in item 5.4, had their IL-1β concentration evaluated. After centrifugation, the supernatant was used for quantification of IL-1β levels by specific Enzyme Immunoassay (EIA), according to Pontes et al. [56]. The results were expressed in pg/mL.

#### Pharmacologic Modulation of P2X7 Receptor

To assess the participation of P2X7 receptor on LDH, CK, and IL-1β liberation, the animals were treated with 50 mM/Kg of A438079, a P2X7 receptor antagonist, 30 min before PBS or BthTX-I inoculation. The inhibitor concentration used did not have adverse effect on cell viability during the assays and was based on comparison with concentrations used elsewhere in the literature. 

### 5.8. Real-time qRT-PCR

For this assay, two groups of five mice (18–20 g) each received an intramuscular injection of 50 µL, in the right thigh, of sterile phosphate-buffered saline (PBS, 14 mM NaCl, 2 mM NaH_2_PO_4_H_2_O, 7 mM Na_2_HPO_4_12H_2_O) pH 7.2 or 50 µL of BthTX-I (25 µg/mL) dissolved in sterile PBS. After 1 and 2 h, mice were euthanized by cervical dislocation and gastrocnemius muscle removed, macerated, and resuspended in 1 mL of Trizol Reagent (Thermo Fisher Scientific) at RNase-free conditions was used for qRT-PCR. The cDNA was transcribed from RNA using SuperScript III RT (Thermo Fisher Scientific). The quantitative qRT-PCR was performed using RotorGene Q (Qiagen). For this, the reactions were performed using the iTaq™ Universal SYBR® Green Supermix (Bio-Rad) kit. Pairs of specific primers for the gene transcriptions (NLRP3, Caspase 1, ASC, IL-1β, IL-6, IL-18, and P2X7R) and β-actin (control) were designed and synthesized (DNA Express) specifically for this reaction (Table 1). The relative fold change quantification of each gene was calculated by the 2ΔΔCt method using the reference β-actin gene for normalization [57]. The efficiency for each set of primer was 100%. All real-time experiments were performed in triplicate of five independent experiments.

### 5.9. Statistical Analysis

The means and S.E.M. of all data were obtained and compared by one-way ANOVA, followed by Tukey test with significance probability levels less than 0.05.

## Figures and Tables

**Table 1:** Primers used for qRT-PCR.

Gene	Sequence	GenBank	Primer Bank
*β-actin*	F: GGCTGTATTCCCCTCCATCG R: CCAGTTGGTAACAATGCCATGT	NM_007393	6671509a1
*ASC*	F: CTTGTCAGGGGATGAACTCAAAA R: GCCATACGACTCCAGATAGTAGC	NM_023258	31560222a1
*Caspase 1*	F: ACAAGGCACGGGACCTATG R: TCCCAGTCAGTCCTGGAAATG	NM_009807	6753282a1
*IL-1β*	F: GCAACTGTTCCTGAACTCAACT R: ATCTTTTGGGGTCCGTCAACT	NM_008361	6680415a1
*IL-6*	F: TAGTCCTTCCTACCCCAATTTCC R: TTGGTCCTTAGCCACTCCTTC	NM_031168	13624311a1
*IL-18*	F: GACTCTTGCGTCAACTTCAAGG R: CAGGCTGTCTTTTGTCAACGA	NM_008360	6680413a1
*NLRP3*	F: ATTACCCGCCCGAGAAAGG R: TCGCAGCAAAGATCCACACAG	NM_145827	22003870a1
*P2X7R*	F: GACAAACAAAGTCACCCGGAT R: CGCTCACCAAAGCAAAGCTAAT	NM_001038845	6754964a1

## Supplementary Materials

The following are available online at https://www.mdpi.com/2072-6651/12/1/22/s1, Supplementary Video S1. Intravital images of gastrocnemius muscle induced by BthTX-I. Leukocyte influx to the gastrocnemius muscle after BthTX-I injection. BthTX-I (25 µg/animal) was injected into gastrocnemius muscle. After 3 h of this injection, the muscle was exposed and lightly pressed against the glass of the experimental plate, and analyzed by intra-vital microscope as described in Materials and Methods. It was possible to observe a large leukocyte influx when compared to the animal injected with PBS (negative control). Supplementary Video S2. Intravital images of gastrocnemius muscle induced by control. Leukocyte influx to the gastrocnemius muscle after pyrogen-free PBS injection (negative control). PBS was injected into gastrocnemius muscle. After 3 h of this injection, the muscle was exposed and lightly pressed against the glass of the experimental plate, and analyzed by intra-vital microscope as described in Materials and Methods. It was possible to observe that PBS did not induce leukocyte influx when compared to the animal injected with BthTX-I. Figure S1: *Western blot* of NLRP3 inflammasome components expressed in gastrocnemius muscle: NLRP3 receptor, Caspase 1 (p10) from BthTX-I-inoculated, A438079-treated and BthTX-I-inoculated groups, and control PBS.
