# Supplementary material for: Inflammasome Activation Induced by a Snake Venom Lys49-Phospholipase A2 Homologue
Source: Toxins (Basel). 2019 Dec 31;12(1):22. doi: 10.3390/toxins12010022 (PMC7020408; doi:10.3390/toxins12010022)
Supplement: Supplementary file 1 [file toxins-12-00022-s001.zip › toxins-664587-supplementary-for conversion.docx]

Inflammasome Activation Induced by a Snake Venom Lys49-Phospholipase A_2_ Homologue

Charles Nunes Boeno, Mauro Valentino Paloschi, Jéssica Amaral Lopes,
Weverson Luciano Pires, Sulamita da Silva Setúbal, Jaína Evangelista Rodrigues,
Andreimar Martins Soares and Juliana Pavan Zuliani


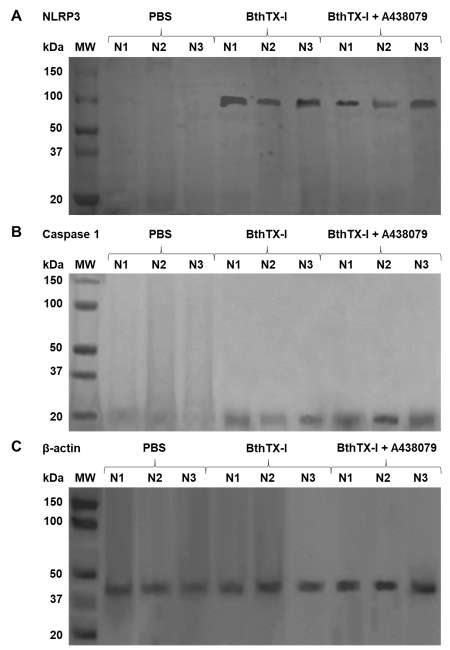


**Figure S1. Protein expression.** Swiss mice were divided into three groups: inoculated with PBS, BthTX-I, and one group inoculated with BthTx-I and treated with P2X7R receptor inhibitor via i.p. (30 minutes after BthTX-I injection into the gastrocnemius muscle). After 3 h of these injections the muscles were removed and processed for NLRP3 inflammasome components protein expression. *Western blot* of NLRP3 inflammasome components expressed in gastrocnemius muscle: NLRP3 receptor, Caspase 1 (p10) from BthTX-I-inoculated, A438079-treated and BthTX-I-inoculated groups, and control PBS. (**A** NLRP3 protein expression); (**B** Caspase-1 p10 protein expression) and (**C** β-actin protein expression). Blots of 3 animals (N = 3). MW: molecular weight.
